# The Two-Species Model of transketolase explains donor substrate-binding, inhibition and heat-activation

**DOI:** 10.1038/s41598-020-61175-z

**Published:** 2020-03-05

**Authors:** Henry C. Wilkinson, Paul A. Dalby

**Affiliations:** Department of Biochemical Engineering, University College London, London, WC1E 6BT UK

**Keywords:** Biocatalysis, Holoenzymes, Biological fluorescence, Post-translational modifications

## Abstract

We recently characterised a low-activity form of *E. coli* transketolase, TK_low_, which also binds the cofactor thiamine pyrophosphate (TPP) with an affinity up to two-orders of magnitude lower than the previously known high TPP-affinity and high-activity form, TK_high_, in the presence of Mg^2+^. We observed previously that partial oxidation was responsible for increased TK_high_ activity, while low-activity TK_low_ was unmodified. In the present study, the fluorescence-based cofactor-binding assay was adapted to detect binding of the β-hydroxypyruvate (HPA) donor substrate to wild-type transketolase and a variant, S385Y/D469T/R520Q, that is active towards aromatic aldehydes. Transketolase HPA affinity again revealed the two distinct forms of transketolase at a TK_high_:TK_low_ ratio that matched those observed previously via TPP binding to each variant. The HPA dissociation constant of TK_low_ was comparable to the substrate-inhibition dissociation constant, *K*_*i*_^*HPA*^, determined previously. We provide evidence that *K*_*i*_^*HPA*^ is a convolution of binding to the low-activity TK_low_-TK_low_ dimer, and the TK_low_ subunit of the partially-active TK_high_-TK_low_ mixed dimer, where HPA binding to the TK_low_ subunit of the mixed dimer results in inhibition of the active TK_high_ subunit. Heat-activation of transketolase was similarly investigated and found to convert the TK_low_ subunit of the mixed dimer to have TK_high_-like properties, but without oxidation.

## Introduction

Transketolase is a key enzyme of the pentose phosphate pathway (PPP), is ubiquitous in all organisms, and provides a unique link between glycolysis and the non-oxidative phase of the PPP. Transketolase is a thiamine pyrophosphate (TPP)-dependent enzyme that reversibly transfers a two-carbon ketol group from a donor substrate (usually a five-carbon ketose) to an acceptor aldehyde substrate (usually a five-carbon aldose) via a ping-pong reaction mechanism, forming a new asymmetric C-C bond with high regio- and stereo-specificity. In biocatalysis, β-hydroxypyruvate (HPA) is often used as the donor substrate due to the irreversible, concomitant release of CO_2_ as a by-product. Strong substrate inhibition has been observed above 25 mM HPA with an inhibition constant of around 42 mM^1,2^. The cause of this substrate inhibition is currently unknown and is addressed in this study.

The inactive, apo-form of transketolase is in a monomer-dimer equilibrium that is dependent on protein concentration. Upon cofactor binding, both the inactive apo-monomer and apo-dimer are converted into the catalytically active, dimeric holo-form of, until recently, seemingly structurally-identical subunits, with two active-sites per homodimer, located at the subunit interface^3,4^. Each active site is comprised of one divalent cation, such as Mg^2+^, and one TPP molecule. In the S. cerevisiae transketolase apo-dimer, even after removal of free Ca^2+^, one Ca^2+^ ion was bound extremely tightly to one active site and could only be removed using harsh treatment, while the second Ca^2+^ ion dissociated easily. Subsequent addition of Mg^2+^ to the medium led to a transketolase dimer with one Ca^2+^- and one Mg^2+^-reconstituted active site, with positive cooperativity observed between active sites upon TPP-binding^5^. It is unclear if *E. coli* transketolase behaves in a similar way, since both positive and negative cooperativity have been observed at different [Mg^2+^]^6^.

Transketolase displays ping-pong kinetics and catalyses two sequential half-reactions; formation of the dihydroxyethyl-TPP (DHE-TPP) carbanion intermediate, followed by transfer of this two-carbon ketol group from the carbanion intermediate to an acceptor aldehyde, thus returning the enzyme to its starting state. It is thought that there is considerable communication between active sites in order to coordinate the ping-pong kinetics between active sites, such that catalysis alternates between active sites, giving rise to the ‘half-of-the-sites reactivity’ phenomenon that has been observed in transketolase^7^ and other TPP-dependent enzymes^8–11^.

There is evidence that in the E1 subunit of the pyruvate dehydrogenase complex (PDC) (EC 1.2.4.1), the two TPP-containing active-sites communicate via a 20 Å proton wire that shuttles a proton between active sites, enabling the cofactors to operate reciprocally as general acid-base catalytic moieties, thus synchronising the ping-pong mechanism across active sites^12^. It was suggested by the authors that many thiamine-dependent enzymes may function in a similar way. Recently, the expected structural asymmetry between the two monomers of apo-dimeric *E. coli* TK was finally observed at the proton scale and was implicated as a key feature of the proton wire and hence cooperativity between active sites^13^.

Transketolase is unusual in that incubation at between 40–55 °C for 1 h increases the residual activity significantly, when measured after re-cooling the samples to 25 °C. For example, incubation at 42 °C for 1 hr increases activity by 50%^14,15^. It was postulated that this curious phenomenon may be the result of an inactive form of transketolase being physically altered or activated by temperature, although no such species had been detected at that time.

Until recently, it was thought that purified transketolase existed in a single form with a high affinity for TPP, which is essential for catalytic activity. However, we previously found that transketolase exists with two distinct subpopulations of subunits in purified samples, TK_high_ and TK_low_, with over 200-fold different affinities for TPP at high [Mg^2+^]^6^. TK_high_ was found to form via at least one specific oxidation of the unmodified TK_low_. The TK_low_ subunit had only 4.5% of the activity of TK_high_ in the presence of saturating concentrations of TPP, and was also found to have disrupted the cooperativity between TPP-binding sites observed for TK_high_. The two distinct monomeric subunits were found to combine into at least two distinct dimer forms, the TK_high_-TK_high_ and TK_low_-TK_low_ dimers, while the TK_high_-TK_low_ mixed dimer was not observed directly, but not ruled out as possible.

The fraction of TPP that can bind to TK_high_ relative to TK_low_, %*B*_*max(high*)_^*TPP*^ (33.6 ± 2.9%), was found to be invariant to changes in [Mg^2+^] and [TK], while the addition of thiamine during fermentation to increase cellular [TPP] also had no effect. It was hypothesized that oxidative stress may play a role in determining the ratio of TK_high_:TK_low_, and detection of post-translational oxidations by mass spectrometry in TK_high_ supported our hypothesis^6^.

The pentose phosphate pathway runs parallel to glycolysis and has several cellular functions, including the generation of pentose sugars as well as ribose-5-phoshate, the latter the precursor for nucleotide biosynthesis. It is therefore a key branch-point in the diversion of metabolic flux towards biosynthetic pathways. However, one of the PPP’s most important utilities is to respond to and negate oxidative stress, often due to reactive oxygen species (ROS), through production of an anti-oxidant, NADPH^16^.

Intracellular NADPH is continuously used as a reducing agent to replenish the reduced glutathione pool to protect against oxidative stress and to maintain a stable cellular redox potential^17–19^. Recently, it has been demonstrated that in the short-term, oxidative stress can be mediated by redox-sensitive enzymes in lower glycolysis^20–23^. In these instances, oxidative post-translational modifications (PTMs) can provide regulation with a rapid response time by cysteine oxidation and subsequent diversion of glycolytic flux through the PPP to generate NADPH and nucleotide precursors for DNA repair. Another separate cysteine-based regulatory system that protects against oxidative stress by diverting flux from NAD^+^-dependent ALDS to NADH-producing ALDHs was also recently elucidated^24^. While a few control points have been identified in lower glycolysis^20–23^ and in the oxidative phase of the PPP^16^, to the authors’ knowledge redox regulation of enzymes in the non-oxidative phase of the PPP are yet to be identified or fully characterised. The identification of such regulatory mechanisms may also inform studies into the role of transketolase in tumour progression, given its increased activity found in many cancer cells^25^.

Here, we examine directly the binding of substrate to the transketolase species subpopulations for both wild type and the variant S385Y/D469T/R520Q, and provide further evidence that transketolase is a redox-regulated enzyme, that could therefore potentially play a major role in the control of flux through the PPP during oxidative stress. The new substrate inhibition and heat-activation data indicated that a mixed dimer species, TK_high_-TK_low_, may be the cause of donor-substrate inhibition and heat-activation. Finally, we propose an updated Two-Species Model for transketolase activation, regulation and inhibition that includes explanations of the origin of %*B*_*max(high)*_, HPA inhibition, active-site cooperativity, redox control and heat-shock activation.

## Results

### The Two-species Transketolase model is persistent across variants

**Figure 1:**
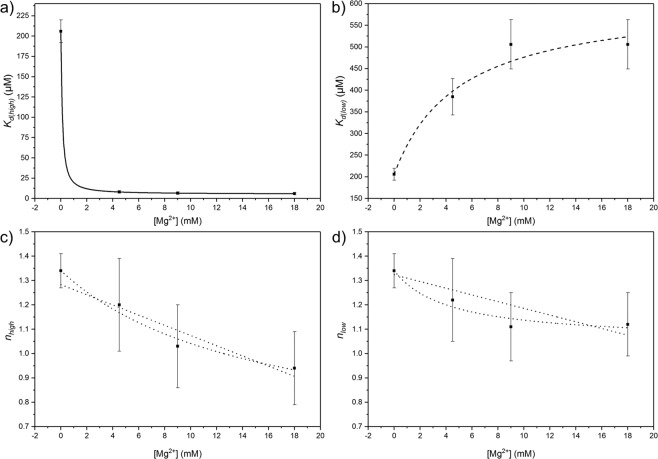
Dependence of TPP-dissociation constants and Hill coefficients of the high and low affinity binding sites for 0.05 mg/mL TK (S385Y/D469T/R520Q) at 0 mM, 4.5 mM, 9 mM and 18 mM Mg^2+^. (**a,b**) – the dissociation constants of the high and low affinity binding sites, respectively. (**c,d**) – the Hill coefficients of the high and low affinity binding sites, respectively. Solid lines represent quantified, fitted graphs; dashed lines represent clear, unquantified trends; dotted lines represent possible trends that fit the data. Associated errors are the fitting error when fitting to the double-Hill equation.

Previously, we measured the TPP-binding parameters of wild-type transketolase over a range of cofactor concentrations^6^. Here, we report the TPP-binding parameters for S385Y/D469T/R520Q, a variant that was previously engineered for activity towards aromatic aldehydes^26^. The rationale behind these measurements was to (a) confirm the existence of both TK_high_ and TK_low_ across variants; and (b) to demonstrate a direct correlation between changes in the % substrate inhibition, *%B*_*max(high)*_, and %TK_modified_. The TPP-binding parameters of TK_high_ and TK_low_ for S385Y/D469T/R520Q at between 0–18 mM Mg^2+^ are summarised below (Fig. 1a–d; Tables 1 and 2; Fig. S1, Supplementary Information). The double-Hill function^6^ was again utilised to determine the independent TPP-binding parameters for TK_high_ and TK_low_. As previously for wild-type TK^6^, at 0 mM Mg^2+^, the binding parameters of TK_high_ and TK_low_ for S385Y/D469T/R520Q were too similar to deconvolve with accuracy (Fig. S2, Supplementary Information), therefore the dissociation constants and Hill coefficients were constrained to equal each other within the double-Hill function.

**Table 1:** Summary of the TPP-binding parameters of the high affinity binding sites of the TK_high_ species of S385Y/D469T/R520Q.

[Mg^2+^] (mM)	*K*_*d(high)*_^*TPP*^ (µM)	±	*n*_*high*_^*TPP*^	±
0	206	14	1.34	0.07
4.5	8.03	1.59	1.20	0.19
9	6.60	1.64	1.03	0.17
18	5.96	1.52	0.94	0.15

A TK concentration of 0.05 mg/mL was used in each binding assay. Associated errors are the fitting error when fitting to the double-Hill function.

**Table 2:** Summary of the TPP-binding parameters of the low affinity binding sites of the TK_low_ species of S385Y/D469T/R520Q.

[Mg^2+^] (mM)	*K*_*d(low)*_^*TPP*^ (µM)	±	*n*_*low*_^*TPP*^	±
0	206	14	1.34	0.07
4.5	385	42	1.22	0.17
9	506	57	1.11	0.14
18	506	57	1.12	0.13

A TK concentration of 0.05 mg/mL was used in each binding assay. Associated errors are the fitting error when fitting to the double-Hill function.

The trends in the TPP-binding parameters of TK_high_ and TK_low_ of S385Y/D469T/R520Q were similar to those of wild-type transketolase^6^. A significant decrease and comparatively small increase in the dissociation constants were observed for TK_high_ (Fig. 1a) and TK_low_ (Fig. 1b), respectively, at higher [Mg^2+^]. However, TK_high_ and TK_low_ of S385Y/D469T/R520Q each bound to TPP with a lower affinity than the wild-type (4.5-fold and 1.6-fold lower at 18 mM Mg^2+^, respectively), and TPP-binding to TK_high_ (Fig. 1c) was less cooperative in S385Y/D469T/R520Q. Furthermore, the binding cooperativity changed from being positive, to non-cooperative as [Mg^2+^] was increased, but no longer peaked at the physiologically relevant [Mg^2+^] of 4 mM as observed in wild-type TK^6^. The trends were most likely non-linear in nature, but a linear relationship could not be ruled out (Fig. 1c,d). These results indicated that while changes in maximum affinity may change between variants, the response of TK_high_ and TK_low_ to Mg^2+^ was persistent across variants, although the cooperativity was impacted quite considerably.

### Adaption of the fluorescence quenching-based TPP-binding assay for donor substrate binding

Similar to our previous fluorescence quenching-based cofactor-binding assay^6^, binding of HPA to holo-transketolase was shown to further quench the intrinsic fluorescence of holo-transketolase (λ_ex_ = 240 nm; λ_em_ = 330 nm) (Fig. S3, Supplementary Information). Like TPP, HPA absorbed relatively strongly at 240 nm. The sample signal was therefore also corrected for the inner filter effect (IFE) by generating a correction factor, determined empirically from the change in fluorescence intensity of 0–80 mM free HPA in 50 mM Tris-HCl buffer, 9 mM Mg^2+^ and 0.3 mM TPP (Fig. S4, Supplementary Information).

### Characterisation of *E. coli* transketolase binding to HPA

**Figure 2:**
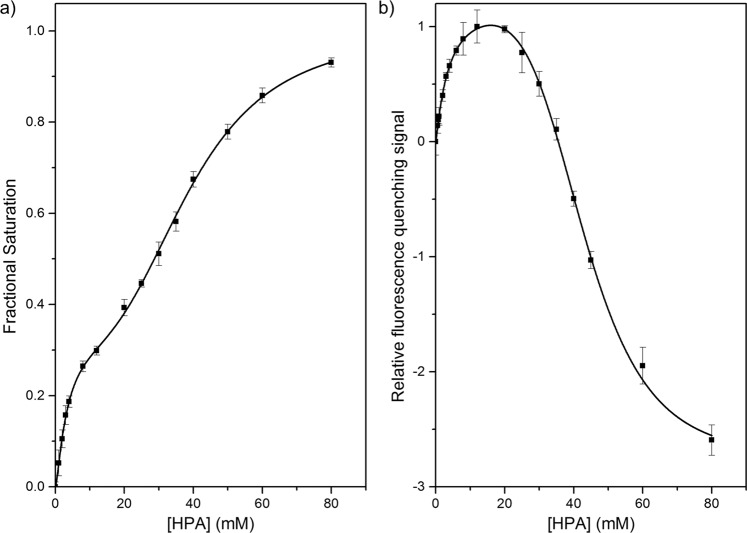
Experimental data of 0.05 mg/mL (**a**) wild-type and (**b**) S385Y/D469T/R520Q transketolase binding to HPA in the presence of 9 mM Mg^2+^ and 0.3 mM TPP in 50 mM Tris-HCl buffer. Experimental data was fitted to the double-Hill function^6^. The change in binding was reported as the ‘relative fluorescence quenching signal’ in (**b**) because of the change in signal from fluorescence quenching to increased fluorescence at above 20 mM HPA. As such, normalisation to give fractional saturation is not possible in (**b**). Error bars correspond to the standard error of the mean, n = 5.

The dissociation constant of HPA-binding to transketolase, *K*_*d*_^*HPA*^, has never been previously determined directly, although the closely-related Michaelis-Menten constant of HPA-binding to wild-type transketolase, *K*_*m*_^*HPA*^, has been reported as 5.5 ± 0.5 mM^27^ and 5.3 mM^28^. Substrate inhibition by HPA, *K*_*i*_^*HPA*^, has also been measured as 42.2 mM and 43 mM^1,2^, providing suitable benchmarks for comparison. The double-Hill function^6^ was again used to determine the HPA-binding parameters for both wild-type transketolase and S385Y/D469T/R520Q, as the fit to the data was superior to a single Hill function (Fig. 2a–b; Table 3), as reported previously^6^.

**Table 3:** Summary of the binding parameters of the high and low affinity binding sites of TK_high_ and TK_low_ of wild-type and S385Y/D469T/R520Q transketolase.

Variant	*K*_*d(high)*_^*HPA*^ (mM)	±	*K*_*d(low)*_^*HPA*^ (mM)	±	*n*_*high*_^*HPA*^	±	*n*_*low*_^*HPA*^	±
Wild-type	3.42	0.45	39.3	0.7	1.39	0.19	3.14	0.18
S385Y/D469T/R520Q	3.61	0.31	43.1	0.4	1.15	0.06	4.60	0.21

Each binding assay used 0.05 mg/mL transketolase, 9 mM MgCl_2_ and 0.3 mM TPP in 50 mM Tris-HCl buffer. Associated errors are the fitting error when fitting to the double-Hill function.

**Figure 3:**
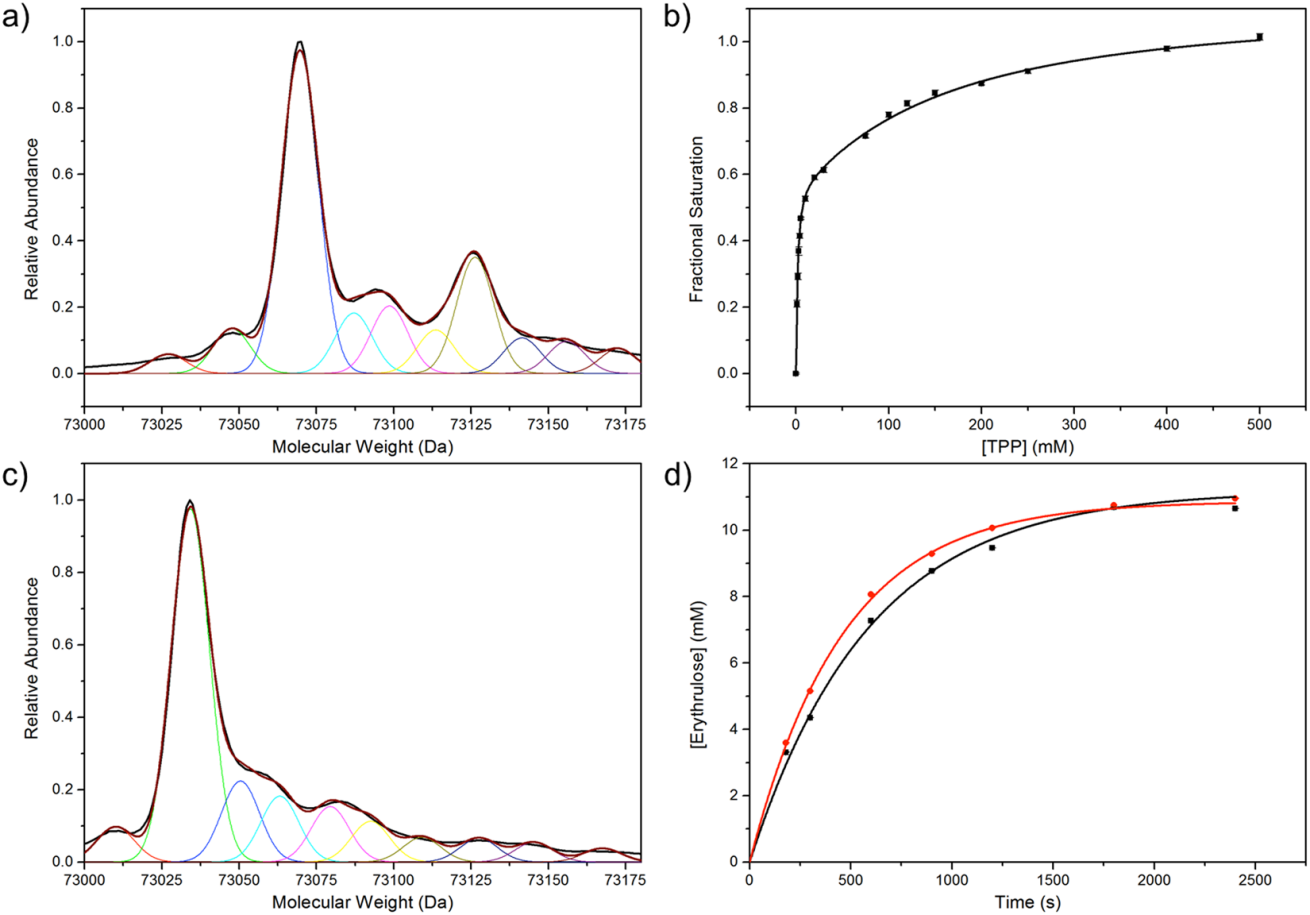
The impact of heat-activation on %TK_high_, TPP-binding and activity. The mass spectra of purified (**a**) S385/D469T/R520Q and (**c**) heat-activated wild-type TK (1 hr at 42 °C). The major peak corresponded to unmodified TK, while the next two peaks corresponded to modified TK^6^. (**b**) TPP-binding to heat-activated 0.05 mg/mL wild-type TK (1 hr at 42 °C), 9 mM Mg^2+^. Error bars correspond to the standard error of the mean, n = 5. (**d**) Activity data of purified 0.067 mg/mL wild-type TK with 50 mM GA and 50 mM HPA, pre-incubated with 9 mM Mg^2+^ and 50 µM TPP before (black) and after (red) heat-activation (1 hr at 42 °C). Error bars correspond to the standard error of the mean, n = 3.

Two distinct HPA-binding events, TK_high_ and TK_low_, were detected, with an 11-fold difference in affinity, supporting our previously postulated Two-Species Model of transketolase. The three mutations within the S385Y/D469T/R520Q variant appeared to have no significant effect on the affinity of HPA binding, but significantly decreased the cooperativity of both TPP- and HPA-binding in TK_high_, and increased the cooperativity of HPA-binding in TK_low_ (Table 3). In addition, binding of HPA to TK_low_ resulted in a decrease in fluorescence quenching in S385Y/D469T/R520Q. This was presumably related to the introduction of the fluorescent tyrosine at residue 385. Importantly, the proportion of HPA bound to TK_high_ relative to all TK at saturation, %*B*_*max(high)*_^*HPA*^, matched that for TPP bound to TK_high_, %*B*_*max(high)*_^*TPP*^, for each variant (Tables 4 and 5), indicating that the structural difference between TK_high_ and TK_low_ impacted both TPP and HPA binding, and confirmed that the two species remained distinct from each other. Furthermore, the %*B*_*max(high)*_^*HPA*^ and %*B*_*max(high)*_^*TPP*^ also matched the %TK_modified_ determined from the mass spectra of wild-type transketolase^6^ and the variant S385Y/D469T/R520Q (Fig. 3a; Table 4), demonstrating that the enzyme affinity parameters and the oxidation to form TK_high_ were correlated.

**Table 4:** The %*B*_*max(high)*_^*TPP*^ and %*B*_*max(high)*_^*HPA*^ and %TK_modified_ of wild-type transketolase^6^ and variant S385Y/D469T/R520Q, determined by TPP-binding, HPA-binding and mass spectrometry data.

Variant	%*B*_*max(high)*_^*TPP*^	±	%*B*_*max(high)*_^*HPA*^	±	%TK_modified_	±
Wild-type	33.6%	2.9%	33.7%	3.0%	31.0%	1.7%
S385Y/D469T/R520Q	27.1%	5.3%	23.8%	5.0%	30.0%	0.1%

Associated errors are either the the fitting error (%*B*_*max(high)*_^*TPP*^ and %*B*_*max(high)*_^*HPA*^) or the standard error of the mean (%TK_modified_, n = 3).

**Table 5:** Summary of the TPP-dissociation constants of wild-type TK_low_ and TK_high_ pre- and post-incubation at 42 °C for 1 h.

Sample	*K*_*d(high)*_ (µM)	±	*K*_*d(low)*_ (µM)	±	%*B*_*max(high)*_	±
Wild-type	2.28	0.23	276	23	33.6%	2.9%
Heat-activated wild-type	1.75	0.26	157	28	51.7%	5.1%

A TK concentration of 0.05 mg/mL was used in each binding assay. Associated errors are the fitting error when fitting to the double-Hill equation.

The *K*_*d*_^*HPA*^ of wild-type TK_high_, *K*_*d(high)*_^*HPA*^, was slightly lower than the previously reported *K*_*m*_^*HPA *^^27,28^. This was expected because *K*_*m*_ is measured via enzyme kinetics, and is therefore a chemical pseudo-equilibrium which is convoluted with the additional forward reaction for formation of product, in other words substrate turnover (*k*_*cat*_). By comparison, *K*_*d*_^*HPA*^ was obtained as a direct equilibrium measurement for binding of HPA. The *K*_*d*_^*HPA*^ of wild-type TK_low_, *K*_*d(low)*_^*HPA*^, was very similar to the previously reported inhibition constant of HPA, *K*_*i*_^*HPA*1,2^, which suggested a possible relationship between TK_low_ binding to HPA and substrate inhibition by HPA, despite the fact that the *K*_*i*_^*HPA*^ was obtained with no knowledge of the two-species model of transketolase.

HPA appeared to bind to the wild-type TK_high_ active-sites with slight positive cooperativity (*n* = 1.38 ± 0.19), while HPA binding to TK_low_ was highly cooperative with a Hill coefficient of 3.10 ± 0.16. This high Hill coefficient suggests a potentially important role of TK_low_ inhibition by HPA in the regulation of TK activity.

### Heat-induced activation and conversion of TK_low_ to a TK_high_-like state

The previous study into heat-activation of TK used cofactor concentrations of 0.5 mM Mg^2+^ and 50 μM TPP, rather than 9 mM Mg^2+ ^^14^. At these concentrations, the [TPP] was semi-saturating for TK_high_ (approximately 60% saturated) but too low for TK_low_ saturation (approximately 20% saturated)^6^. In other words, the majority of TK_high_ was in the catalytically active holo-form while TK_low_ was mostly in the catalytically inactive apo-form. Therefore, we hypothesised that heat exposure may convert TK_low_ to TK_high_, hence increasing the concentration of holo-transketolase and overall activity of the sample.

We tested our hypothesis by taking fluorescence quenching measurements of 0.05 mg/mL TK, 9 mM Mg^2+^ and a range of [TPP] before^6^ and after incubation at 42 °C for 1 hour (Fig. 3b; Table 4). As hypothesised, the %*B*_*max(high)*_ increased from 33.7% to 51.7% after heat-activation. In addition, the affinity of both TK_high_ and TK_low_ increased significantly. Performing the same heat activation for a second time on the same sample increased the %*B*_*max(high)*_ only slightly to 53.5% (Fig. S5, Supplementary Information), indicating that no further change could be induced. Conversely, heat-activation had negligible impact on %TK_modified_ (29.4 ± 4.1%), determined from the mass spectra of heat-activated wild-type transketolase (Fig. 3c). Taken together, these results suggested heat-activation may occur through formation of a TK_high_-like conformational state but via a different mechanism to that of oxidation of TK_low_ to TK_high_.

The activity of 0.05 mg/mL TK, 9 mM Mg^2+^ and 50 μM TPP towards 50 mM GA and 50 mM HPA pre- and post-incubation was determined to calculate the activity of heat-activated TK_high_ relative to pre-incubated TK_high_ (Fig. 3d; Table 5). Overall, a 24.6% increase in transketolase activity was observed after heating, consistent with our hypothesis, though approximately only 50% of the activity enhancement expected from the %*B*_*max(high)*_ increase. This may indicate lower activity in the heat-induced TK_high_-like state compared to the oxidised TK_high_, or some partial unfolding and inactivation during heating. These and other possible mechanisms were not investigated further here.

### The existence of a TK_high_-TK_low_ mixed dimer species that mediates HPA substrate inhibition

The equivalence between the *K*_*d(low)*_^*HPA*^ and the *K*_*i*_^*HPA*^ measured in previous activity assays suggested that binding of HPA to TK_low_ gave rise to the observed overall inhibition of transketolase activity. However, the activity of TK_low_ was already only 4.5% relative to that of TK_high_^6^, implying that the TK_low_-TK_low_ dimer was effectively inactive already, and so binding of HPA to that dimer species could not have contributed significantly to the observed reaction inhibition. Therefore, the interaction between HPA and TK_low_ must inhibit the TK_high_ activity, which in turn suggested that inhibition occurred within a TK_high_-TK_low_ mixed dimer form. We therefore attempted to estimate the relative proportions of the three dimeric species, TK_high_-TK_high_, TK_high_-TK_low_, and TK_low_-TK_low_, from the heat-activation, HPA-binding, and enzyme activity data available.

#### Estimation of the % dimer forms from heat-activation data

We first assumed that conversion of TK_low_ to a TK_high_-like state via heat-activation was only possible for the proportion of TK_low_ present within a TK_high_-TK_low_ mixed dimer. We assumed all TK_low_ subunits of the mixed dimer were completely converted to a TK_high_-like state, and calculated the relative proportions of each dimeric species before heat-activation from the change in %*B*_*max(high)*_ upon heat-activation:

$$({\rm{A}})\, \% {{\rm{TK}}}_{{\rm{high}}}-\,{{\rm{TK}}}_{{\rm{low}}}=( \% {{B}_{max(high)}}^{heated}- \% {{B}_{max(high)}}^{unheated})\ast 2=(51.7 \% -33.6 \% )\ast 2=36.2 \% ;$$

$$({\rm{B}})\, \% {{\rm{TK}}}_{{\rm{high}}}-{{\rm{TK}}}_{{\rm{high}}}= \% {{B}_{max(high)}}^{unheated}-\,({\rm{A}})/2=33.6 \% -(36.2/2)=15.5 \% ;$$

$$({\rm{C}})\, \% {{\rm{TK}}}_{{\rm{low}}}-{{\rm{TK}}}_{{\rm{low}}}=100 \% -\,({\rm{A}})\mbox{--}({\rm{B}})=100 \% -36.2 \% -15.5 \% =48.3 \% .$$

#### Estimation of the % dimer forms from HPA-inhibition data

Previous studies into the inhibition of transketolase determined the inhibition constants and the maximum inhibition of wild-type transketolase activity by HPA, *%I*_*max*_, for wild-type (*K*_*i*_^*HPA*^ = 43 mM; *%I*_*max*_ = 48.1 ± 5.1%)^2^ and also the D469T variant of transketolase (*K*_*i*_^*HPA*^ = 40 mM; *%I*_*max*_ = 46.8 ± 11.5%)^29^.

Making the assumption that HPA-binding to the TK_low_ subunit of the mixed dimer resulted in total inhibition of the TK_high_ subunit in that mixed dimer, and that TK_high_ accounted for 91% of total activity, we predicted the relative proportions of the three dimeric species:

$$({\rm{A}})\, \% {{\rm{TK}}}_{{\rm{high}}}-{{\rm{TK}}}_{{\rm{low}}}=( \% {{B}_{max(high)}}^{unheated}\ast ( \% {I}_{max}/91 \% )\ast 2=\,(33.6 \% \ast (48.1 \% /91 \% )\ast 2=35.5 \% $$

$$({\rm{B}})\, \% {{\rm{TK}}}_{{\rm{high}}}-{{\rm{TK}}}_{{\rm{high}}}= \% {{B}_{max(high)}}^{unheated}-\,({\rm{A}})/2=15.8 \% ;$$

$$({\rm{C}})\, \% {{\rm{TK}}}_{{\rm{low}}}-{{\rm{TK}}}_{{\rm{low}}}=100 \% -({\rm{A}})\mbox{--}({\rm{B}})=100 \% -35.5 \% -15.8 \% =48.7 \% .$$

The similarity between the relative proportions of the three dimer species, calculated from the *%I*_*max*_ obtained through activity data, and from TPP-binding data after heat-activation (Table 6), provides compelling evidence for our reasoning above that HPA binding to the TK_low_ subunit within the mixed dimer resulted in inhibition of the associated TK_high_ subunit in that mixed dimer. It also implies that heat-activation may happen via the removal of the donor substrate inhibition when the TK_low_ subunit in the mixed dimer is converted into a TK_high_-like state. The fact that inhibition via the mixed dimer species persisted into a single-mutant TK variant, and with a different acceptor substrate, also suggested that this phenomenon is fundamental to the TK structure and mechanism when utilising HPA as the donor substrate.

**Table 6:** Summary of the predicted % dimer of TK_high_-TK_high_, TK_high_-TK_low_ and TK_low_-TK_low_, calculated from TPP-binding data after heat activation and HPA-inhibition data.

Dimer species	% Species (heat-activation data)	% Species (inhibition data)
TK_high_-TK_high_	15.5%	15.8%
TK_high_-TK_low_	36.2%	35.5%
TK_low_-TK_low_	48.3%	48.7%

Note that the % species from the HPA inhibition data accounts for TK_high_ having only 91% of total activity.

### The unified two-species model of transketolase activation, regulation and inhibition

**Figure 4:**
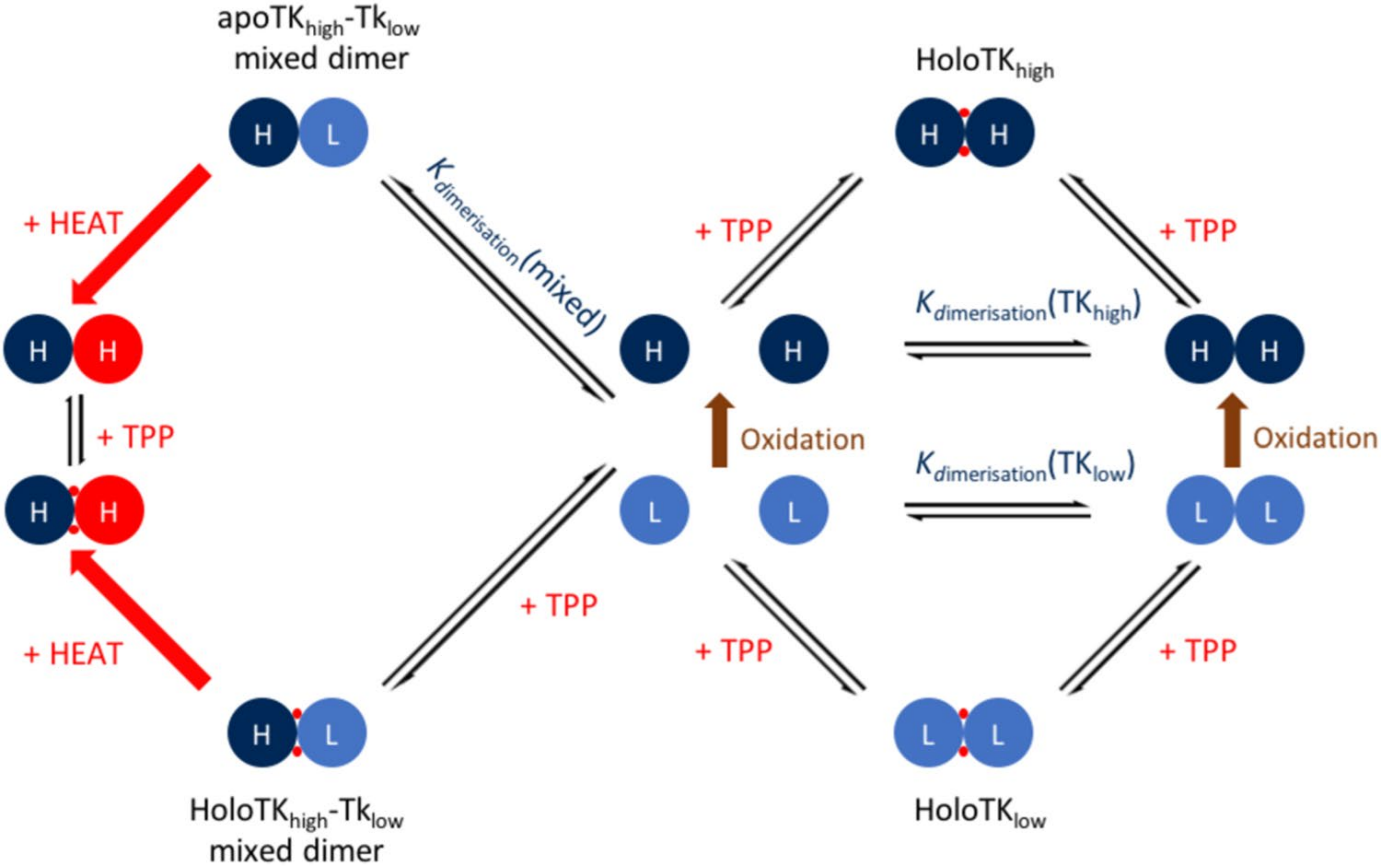
The updated Two-Species Model of transketolase activation, regulation and inhibition. The model is based on the combined TPP binding and AUC data before and after heat activation, as well as activity data. L (light blue) represents an inactive TK_low_ monomer, H (navy blue) an active TK_high_ monomer, and H (red) a TK_high_-like monomer post-heat activation. The mechanism of conversion of TK_low_ to TK_high_ occurs through oxidation of Cys157 (brown).

The development of assays capable of detecting both TK_high_ and TK_low_ has facilitated investigations into donor substrate inhibition, active site cooperativity and heat-activation, and finally led to the discovery of a novel mechanism of transketolase regulation. This can be summarised in the unified Two-Species Model of transketolase activation, regulation and inhibition (Fig. 4).

In summary:

- Transketolase exists as two distinct transketolase species; TK_high_ and TK_low_.
- Inactive TK_low_ is the reduced, unmodified form of transketolase that is converted to TK_high_ via oxidation of one or more methionine or cysteine residues, in response to cellular oxidative stress.
- TK_high_ is significantly more active and also has a 35-fold increased affinity for TPP at physiologically relevant [Mg^2+^].
- Oxidation improves the cooperativity between active sites. Cys157 oxidation is a strong candidate^6^, possibly by providing the final residue of a proton wire between active sites.
- Redox regulation of transketolase potentially provides an important mechanism of control in diverting flux from glycolysis to the PPP during oxidative stress.
- Heat-activation of transketolase converts the TK_low_ subunit of the mixed dimer to a TK_high_-like state, which relieves the substrate inhibition of the associated TK_high_ subunit.
- Heat-activation may offer cells a degree of heat shock protection by activating transketolase - a key enzyme in central metabolism - without the energetic or time-cost of protein biosynthesis.

## Discussion

In this study, we attempted to resolve several unanswered questions remaining from our previous study into transketolase activation^6^; does the two-species phenomenon persist across variants and from cofactor to donor substrate; what is the cause of substrate inhibition; and what is the origin and physiological relevance of heat-activation?

Though wild-type, D469T and S385Y/D469T/R520Q all exist as a mix of TK_high_ and TK_low_, the susceptibility of TK_low_ to oxidation could potentially be enhanced through protein mutations in order to maximise [TK_high_] and hence activity. Alternatively, controlled oxidation of purified transketolase samples may be possible, but this may require careful control to avoid over-oxidation of Cys157, or at other sites, that eventually would cause inactivation.

It is worth noting that this study and our previous study^6^ only investigated TPP binding in the presence/absence of Mg^2+^ and not Ca^2+^. It is possible that Ca^2+^-reconstituted transketolase is activated differently via different oxidation-activation pathways. However, all mass spectra in these studies were derived from transketolases in their apo-form and the formation of modified, oxidised species were hence cation-independent. It is both possible that Ca^2+^-reconstituted transketolase is activated in a similar way to Mg^2+^-constituted transketolase, or active site coordination with Ca^2+^ bypasses the requirement of oxidation for activation. Further studies into TPP- and HPA binding to TK_high_ and TK_low_ in the presence of Ca^2+^ would be able to determine which hypothesis is correct, but are beyond the scope of this study.

It is interesting that HPA binding to TK_low_ had such a high degree of positive cooperativity, and may indeed result from multiple HPA molecules binding to a single active-site to inhibit activity in that active site. Furthermore, it may also inhibit active-site synchronisation by inhibiting the shuttling of protons along the proton wire. The existence of the TK_high_-TK_low_ mixed dimer potentially complicates further the analysis of Hill coefficients for TK_high_ and TK_low_. However, the true Hill-coefficient of TK_high_ is likely to be higher than the reported apparent value, whereas the true Hill-coefficient of TK_low_ is presumably lower. The most abundant dimer, TK_low_-TK_low_, may also have previously masked the recently-elucidated asymmetric structure of the TK apo-dimer^13^, given the reduced cooperativity of TPP-binding between active sites in this dimer.

The heat-activation of TK appeared to increase activity through a slightly different mechanism to that of oxidation of TK_low_ to TK_high_. Conformational rearrangements might result from heat-activation and give rise to a TK_high_-like conformational state that apparently relieves HPA inhibition of the TK_high_ subunit of the mixed dimer. The heat-activation of transketolase may even be part of the cellular response to thermal stress, resulting in the upregulation of the PPP and hence NADPH production, while also increasing flux through critical biosynthetic pathways such as nucleotide biosynthesis. The heat-sensitivity of transketolase therefore has a potential role in facilitating rapid global changes in metabolism without the additional energetic burden of protein synthesis.

Finally, the discovery of a redox-sensitive regulatory system that activates transketolase, potentially during oxidative stress, could have important implications for future advances in cancer treatments. Cancer cells are often exposed to higher levels of oxidative stress than normal cells, and elevated transketolase activity has been reported in a number of cancer types in order to reduce ultimately catastrophic damage from high oxidative stress^30^. The redox-sensitivity of transketolase not only makes transketolase itself an antioxidant, but also an important mediator of the biosynthesis of a second anti-oxidant, NADPH. Reversal or prevention of transketolase oxidation at residue Cys157 by drug delivery or gene therapy may offer an effective way to prevent antioxidant production and hence proliferation in cancer cells.

## Methods

### Materials

TPP, MgCl_2_, glycolaldehyde (GA) and Erythrulose [Ery] were purchased from Sigma-Aldrich; Tris-HCl was purchased from VWR International and Guanidine-HCL was purchased from Life Technologies Ltd. HPA was synthesised from bromopyruvic acid and LiOH, as described previously^31^.

### Enzyme preparation

Wild-type transketolase with an N-terminal His6 tag was expressed in *E. coli* XL10-gold cells (Agilent Technologies Ltd) from the plasmid pQR791. The resulting cell pellet was lysed and purified as described previously^32^. Purified transketolase was ultrafiltrated four times using an Amicon Ultra-4 10k MWCO (Millipore, US) centrifugal filter to remove excess imidazole and cofactors and subsequently dialysed overnight at 4 °C in 50 mM Tris-HCl, pH 7.0 to obtain apo-TK. Protein concentration was determined by absorbance at 280 nm in 6 M Guanidine-HCl and 20 mM Sodium Phosphate, pH 6.5. Absorbance was measured using a Nanodrop spectrophotometer (Thermo Fisher Scientific, Wilmington, DE), assuming a monomeric molecular weight of 73035.5 g mol^−1^ and an extinction coefficient (ε) of 92630 L mol^−1^ cm^−1^.

For TPP-binding assays, series of 2x concentrated cofactor solutions were prepared and purified TK was added to a final concentration of 0.05 mg/mL. The samples were incubated at 22 °C for 45 minutes to allow TK-TPP binding to reach equilibrium. For HPA-binding assays, 2x concentrated, purified holoTK was prepared at 0.1 mg/mL TK, 18 mM Mg^2+^ and 0.6 mM TPP and incubated at 22 °C for 45 minutes to allow TK-TPP binding to reach equilibrium. The purified holoTK was added to a series of 2x concentrated HPA solutions and incubated at 22 °C for 10 minutes to allow holoTK-HPA binding to reach equilibrium. For heat-activation studies, TK samples were incubated at 42 °C for 1 hour and subsequently re-equilibrated at 4 °C for 30 minutes and at 22 °C for 30 minutes prior to assays.

### Fluorescence assay to detect TPP binding

TPP-binding was measured using a Fluoromax-4 (Horiba, UK) spectrofluorometer (λ_ex_ = 240 nm; λ_em_ = 330 nm; integration time = 0.1 s; slit width = 8 nm), as described previously^6^.

### Transketolase activity assay

Purified, dialysed apo-transketolase (0.2 mg/mL) was incubated with 2.4 mM TPP and 9 mM Mg^2+^ for 45 minutes at 22 °C. 50 μL was added to 100 μL 150 mM GA, 150 mM HPA, giving final substrate concentrations of 50 mM. The reaction was performed in triplicate at 22 °C in a 96 well plate with shaking at 300 rpm using a Thermomixer Comfort shaker. 10 μL of the reaction was quenched with 190 μL 0.1% trifluoroacetic acid (TFA) after 3, 5, 10, 15, 20, 30, and 40 minutes. Samples were subsequently analysed by a Dionex HPLC system (Camberley, UK) with a Bio-Rad Aminex HPX-87H reverse phase column (300 × 7.8 mm^2^) (Bio-Rad Labs., Richmond, CA, USA), via Chromeleon client 6.60 software, to separate and analyse the change in the concentration of substrate (GA) and product (Ery) over the course of the reaction using the method described previously^29^.

### Fluorescence assay to detect HPA binding

The same methodology was used as the TPP-binding fluorescence assay, except cofactor concentrations were kept constant at 9 mM Mg^2+^ and 0.3 mm TPP, and an IFE CF was generated between 0–80 mM HPA.

### Mass spectrometry (LC-ESI-MS)

LC-MS was performed using an Agilent 1100/1200 LC system connected to a 6510 A QTOF mass spectrometer (Agilent, UK), as described previously^6^. Triplicate fermentations were performed for each sample.

## Supplementary information

Supplementary information.

## Data Availability

The data that support the findings of this study are available from the corresponding author upon reasonable request.
