# Supplementary material for: The Two-Species Model of transketolase explains donor substrate-binding, inhibition and heat-activation
Source: Sci Rep. 2020 Mar 5;10:4148. doi: 10.1038/s41598-020-61175-z (PMC7057962; doi:10.1038/s41598-020-61175-z)
Supplement: Supplementary file 1 — Supplementary information. [file 41598_2020_61175_MOESM1_ESM.pdf]

The two-species model of transketolase explains donor substrate-binding, inhibition and heat-activation

Henry C. Wilkinson*, Paul A. Dalby

SUPPLEMENTARY INFORMATION

1. TPP-binding to S385Y/D469T/R520Q
2. Comparison of experimental TPP-binding data fitted to a single- or double-Hill function
3. Evidence of fluorescence quenching upon HPA-binding
4. Generation of the inner filter effect correction factor for HPA
5. TPP-binding after double-heat-activation

Section 1: *TPP-binding to S385Y/D469T/R520Q*

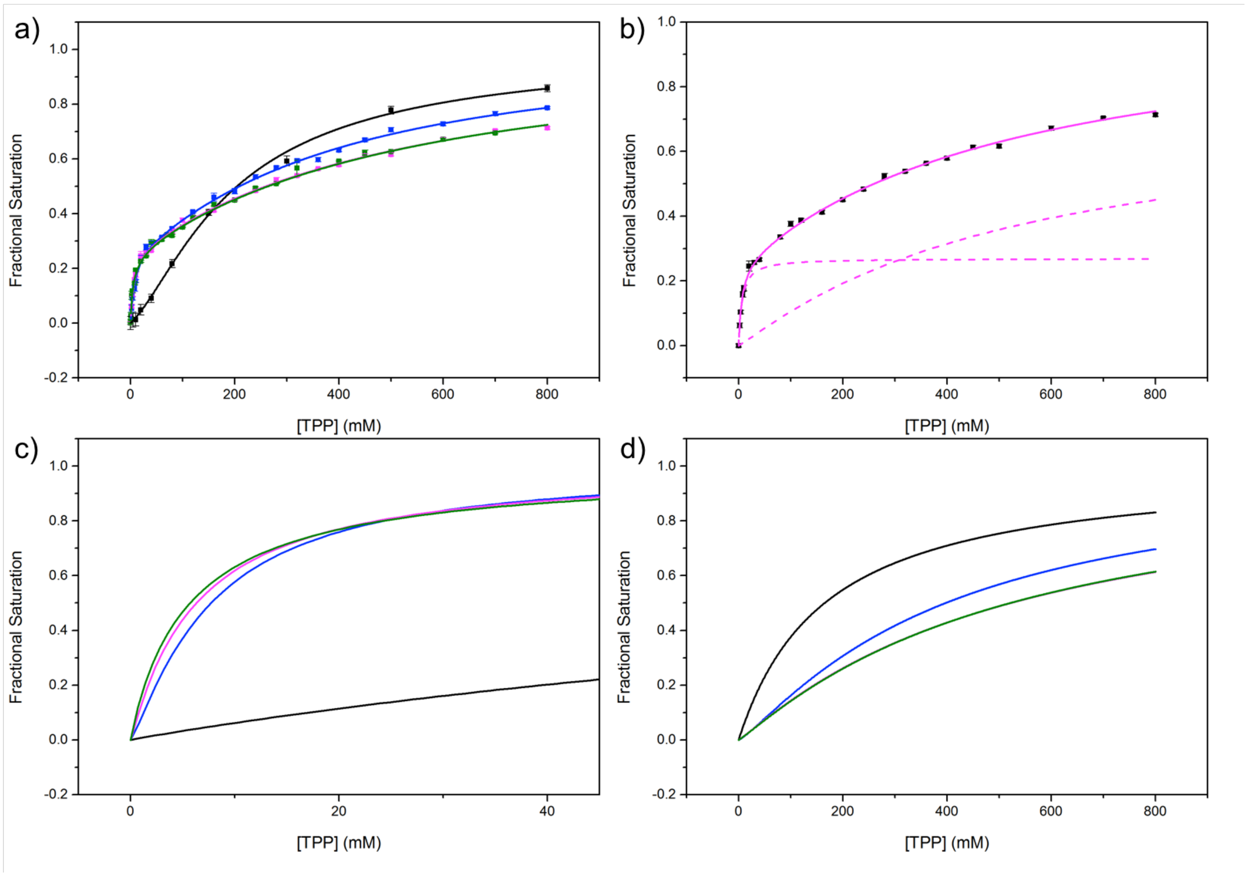

Figure S1: Experimental data of 0.05 mg/mL TK variant S385Y/D469T/R520Q binding to TPP at 0 mM (black), 4.5 (blue), 9 mM (magenta) and 18 mM (green) Mg^{2+}. Experimental data-points a) at all [Mg^{2+}] fitted to the double-Hill function[1]; b) at 9 mM Mg^{2+} (double-Hill function) with the contributions of the high and low affinity binding sites shown as dashed lines; normalised contributions to double-Hill functions, of the c) high affinity and d) low affinity binding sites at each [Mg^{2+}]. For reference, the data in b), c) and d) were derived from the experimental data and corresponding fits plotted in a). Error bars correspond to the standard error of the mean, n=5.

Section 2: *Comparison of experimental TPP-binding data fitted to a single- or double-Hill function*

The double-Hill function[1]: $\theta = \frac{B_{\max(high)} \cdot [L]^{n_{high}}}{K_{d(high)}{}^{n_{high}} + [L]^{n_{high}}} + \frac{\alpha . B_{\max(high)} \cdot [L]^{n_{low}}}{K_{d(low)}{}^{n_{low}} + [L]^{n_{low}}}$

where θ is the fractional saturation, the fraction of [protein] that is bound to ligand; $B_{max(high)}$ is the proportion of TPP that binds to TK_{high}; $\alpha.B_{max(high)}$ is the proportion of TPP that binds to TK_{low}; [L] is the ligand (TPP) concentration; n_{high} and n_{low} are the Hill coefficients of TK_{high} and TK_{low}, respectively; and $K_{d(high)}$ and $K_{d(low)}$ are the dissociation constants of TK_{high} and TK_{low}, respectively.

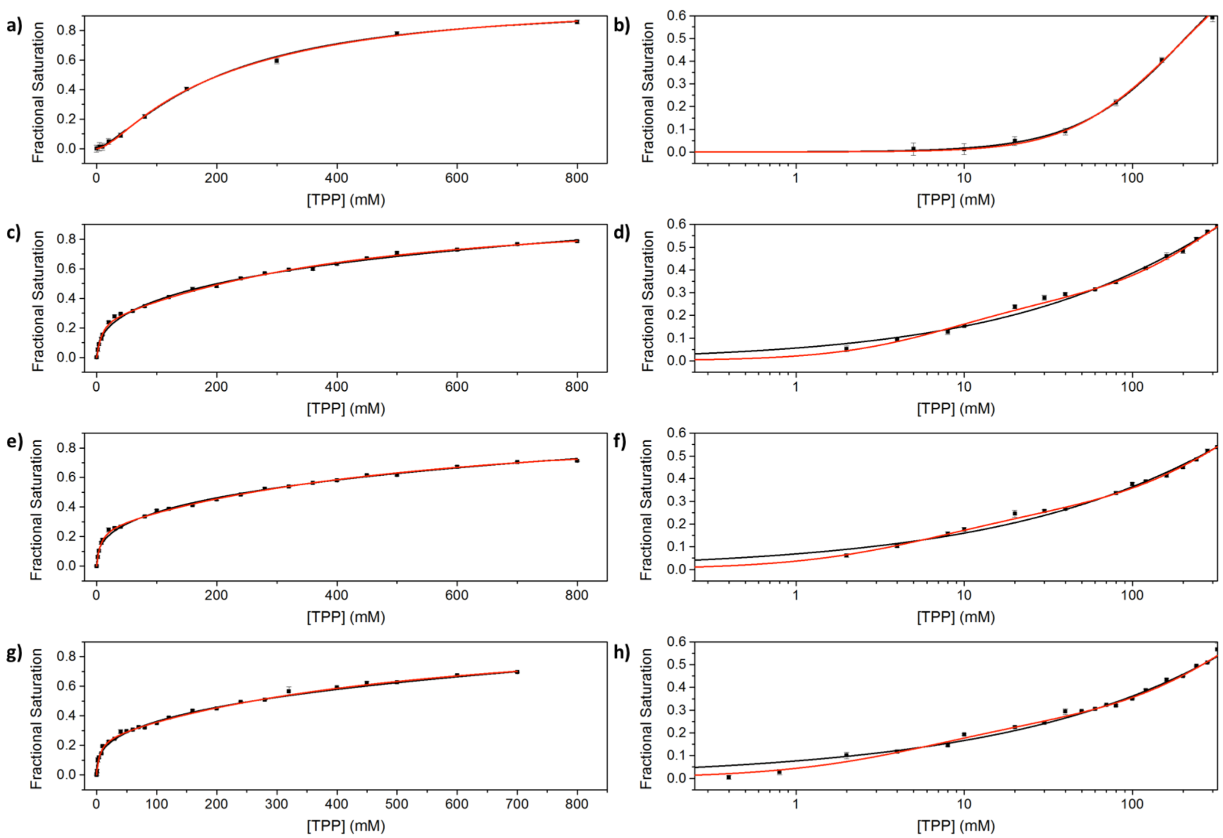

Figure S2: Experimental data of 0.05 mg/mL TK variant S385Y/D469T/R520Q binding to TPP at a) and b) 0 mM; d) and d) 4.5 mM; e) and f) 9 mM; and g) and h) 18 mM Mg^{2+}. Experimental data-points were fitted to either a single- (black) or double- (red) Hill function. The data is presented on a logarithmic x-axis to demonstrate the superior fit to the double-Hill function. Error bars correspond to the standard error of the mean, n=5.

The TPP-binding parameters of TK_{high} and TK_{low} could not be deconvolved at 0 mM Mg^{2+} using the double-Hill function because their values were very similar. Therefore, the dissociation constants and Hill coefficients were constrained to equal each other within the double-Hill function at 0 mM Mg^{2+}. All other TPP-binding parameters determined using the single Hill (Table S1) have no relatability to previously determined dissociation constants and Hill coefficients; at higher [Mg^{2+}], the fits either had very large associated errors or the data couldn't be fitted to the single-Hill function. However, those of TK_{high}, determined by fitting the data to the double-Hill function (Table S2), correlated well with previously reported values. We therefore conclude that we have detected two independent binding events, both cooperative, which fitted best to a double-Hill function.

[Mg^{2+}] (mM)	K_d^{TPP} (μM)	±	n^{TPP}	±
0	206	14	1.34	0.07
4.5	4180	3550	0.45	0.03
9	58100	109000	0.38	0.02
18	-	-	-	-

Table S1: Summary of the TPP-binding parameters when fitted to a single Hill function. A TK variant S385Y/D469T/R520Q concentration of 0.05 mg/mL was used in each binding assay. Associated errors are the fitting error for the single-Hill function. The data couldn't be fitted to the single-Hill function at 18 mM Mg^{2+}.

[Mg^{2+}] (mM)	$K_{d(high)}^{TPP}$ (μM)	±	$n_{(high)}^{TPP}$	±
0	206	14	1.34	0.07
4.5	8.03	1.59	1.20	0.19
9	6.60	1.64	1.03	0.17
18	5.96	1.52	0.94	0.15

Table S2: Summary of the binding parameters of the high affinity binding site, TK_{high}, when fitted to a double-Hill function. A TK variant S385Y/D496T/R520Q concentration of 0.05 mg/mL was used in each binding assay. Associated errors are the fitting error for the double-Hill function.

***Section 3:** Evidence of fluorescence quenching upon HPA-binding*

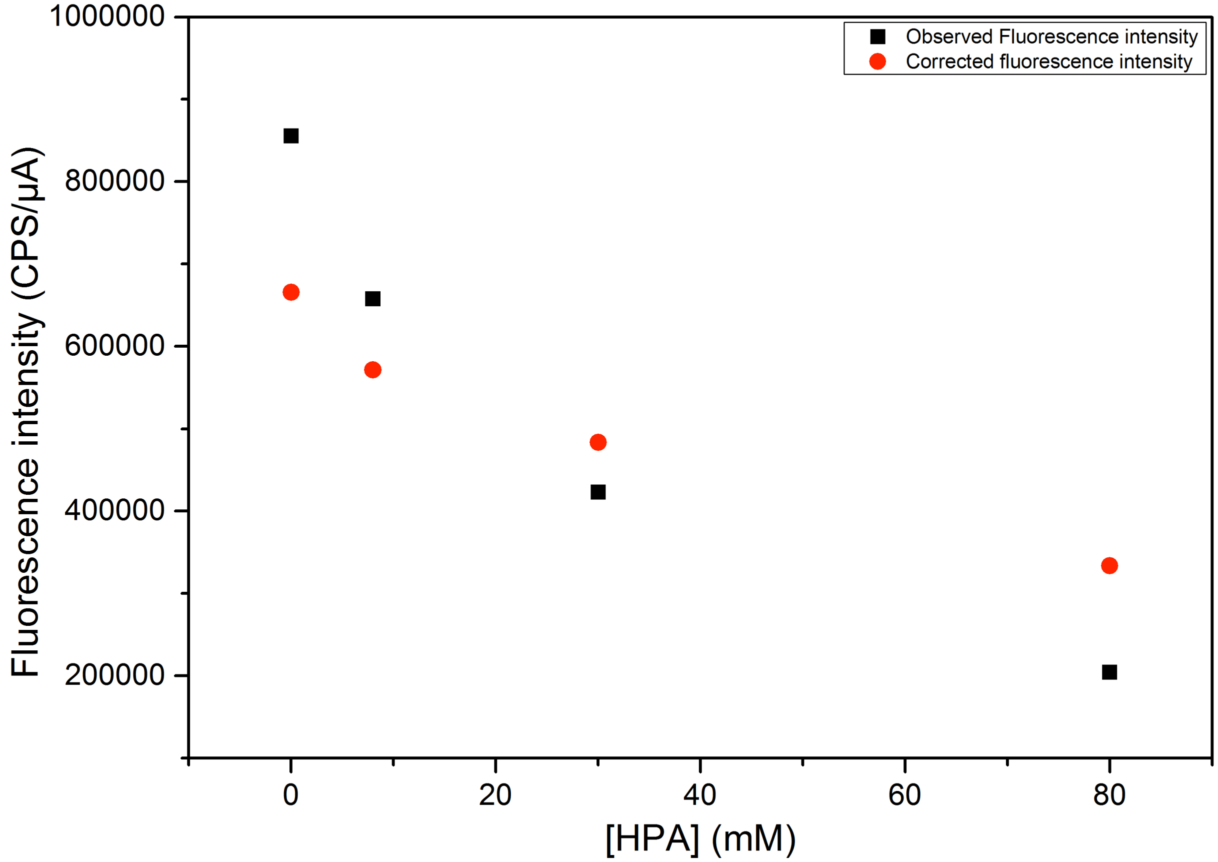

Figure S3: Evidence of fluorescence quenching upon cofactor-binding to wild-type TK. The observed (black) and corrected (red) fluorescence intensity of 0.05 mg/mL TK, 0.3 mM TPP, 9 mM Mg^{2+} and 0-80 mM HPA (λ_{ex} = 240 nm; λ_{em} = 330 nm). Fluorescence intensity was corrected for the inner filter effect (IFE) as described previously[2].

Section 4: *Generation of the inner filter effect correction factor for HPA*

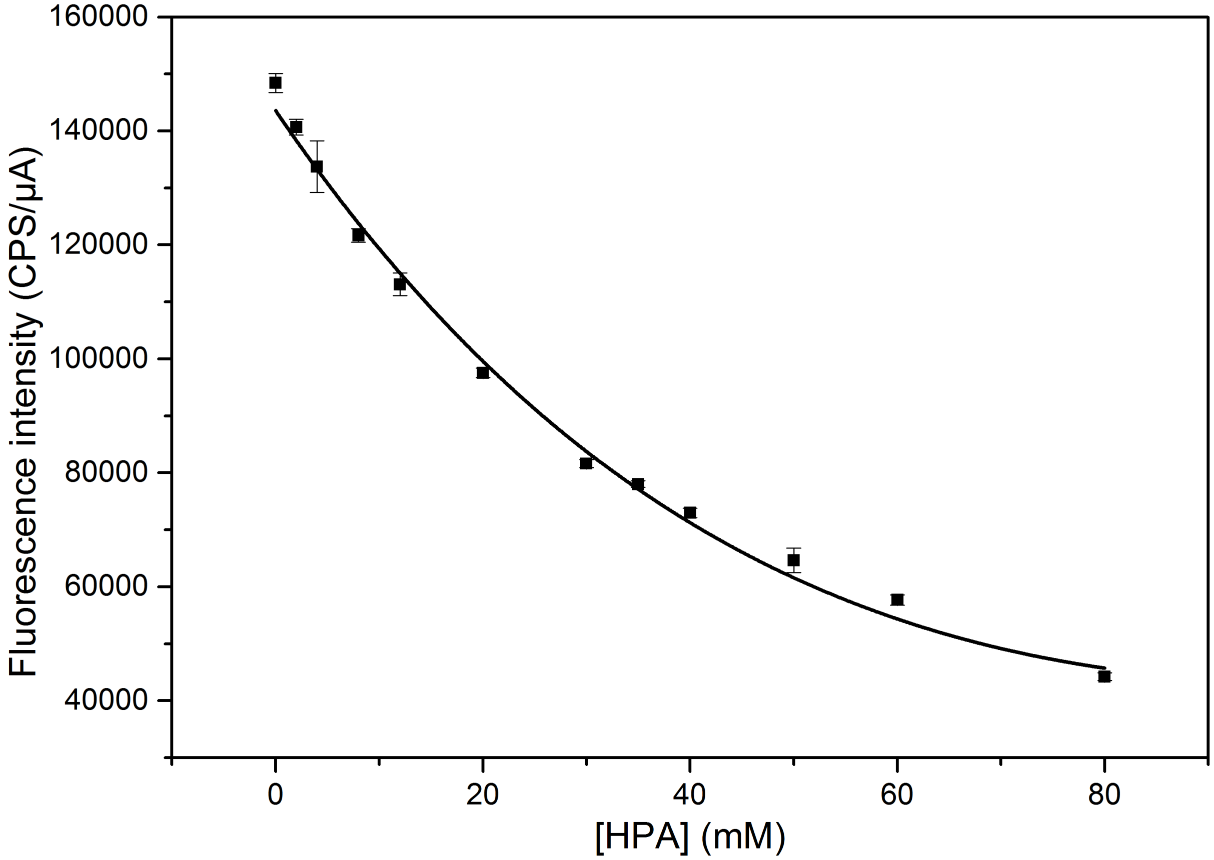

Figure S4: The fluorescence intensity of free HPA in 50 mM Tris buffer, 9 mM Mg^{2+} and 0.3 mM TPP (λ_{ex} = 240 nm; λ_{em} = 330 nm), fitted to the correction function as described previously[2]. Error bars correspond to the standard error of the mean, n=5.

Section 5: *TPP-binding after double-heat-activation*

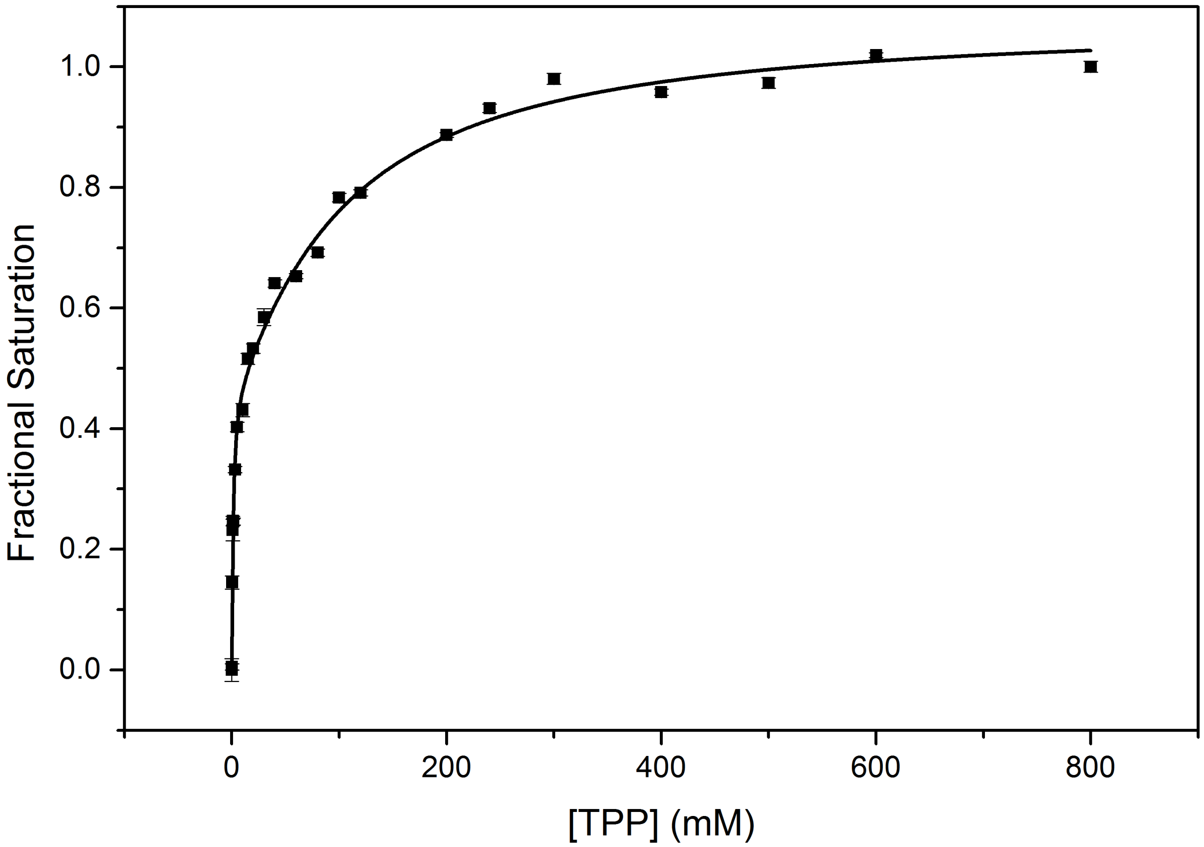

Figure S5: Experimental data of 0.05 mg/mL wild-type TK binding to TPP at 9 mM Mg^{2+}, after two consecutive rounds of heat activation at 42 ºC followed by re-equilibration at 22ºC for 30 minutes. Experimental data-points were fitted to the double-Hill function. Error bars correspond to the standard error of the mean, n=5.

REFERENCES

1. Wilkinson, H. C. & Dalby, P. A. Novel insights into transketolase activation by cofactor binding identifies two native species subpopulations. *Sci. Rep.* **9**, (2019).
2. MacDonald, B. C., Lvin, S. J. & Patterson, H. Correction of fluorescence inner filter effects and the partitioning of pyrene to dissolved organic carbon. *Anal. Chim. Acta* **338**, 155–162 (1997).
